# Research and Remedies

**Published:** 1913-07-26

**Authors:** 


### RESEARCH AND REMEDIES.

### Cerebro-Spinal Fever in Bristol.

One of the physicians to the Bristol Royal Infirmary, Dr. J. R. Charles, writing in the *Bristol Medico-Chirurgical Journal*, states that there can be little doubt that a small epidemic of cerebro-spinal meningitis has been visiting Bristol. Writing in June, there had already been three cases in the Infirmary; and he remarks on the difficulty of diagnosis and the value of lumbar puncture in this connection. All three cases were treated with Flexner's serum, and in the first case the effect appeared to be marvellously beneficial, for the patient was comatose when admitted and recovered rapidly after the serum was administered. The second patient, also unconscious when taken in, died on the day following his admission, and the serum seemed to have no good effect. The third patient was the wife of the second one, and had a milder infection. She was given serum and recovered, but not quite so rapidly as the first patient. In all three cases thick pus was obtained from the spine by lumbar puncture, and the meningococcus was found on microscopic examination. The characteristic petechial eruption (whence the name "spotted fever" for this disease) was absent in all the cases; at least, no mention is made of it. It may be added that urotropin was given as well as Flexner's serum, so that it remains uncertain how much benefit is to be ascribed to the latter remedy and how much to the former.

### Keratitis Neuro-paralytica after Removal of the Gasserian Ganglion.

Two cases of this extremely serious complication are reported in the *New York State Journal of Medicine* by Weidler, who sums up the case for and against "gasserectomy" as an operation for intolerable and intractable neuralgia. The operation is extremely difficult and dangerous, even in the hands of the most eminent surgeons with special experience of cranial surgery; and has a direct mortality of 5 per cent. in the most favourable conditions. It is not infrequently followed by destructive neuro-paralytic keratitis, with ultimate loss of sight and often of the eye. It is quite true that many patients in the throes of trifacial neuralgia are quite willing to sacrifice an eye in the hope of getting relief from pain; and it is admitted that gasserectomy has cured cases which resisted all other treatment. But the author thinks that perfection of the technique of alcohol injection is the best hope of future advance. Already, he says, the patient is assured by this method of relief from pain for a period varying from six months up to many years; and the almost uniform freedom from serious eye complications renders it, in his opinion, the most satisfactory and safest form of treatment. In 300 operations by the alcohol method, keratitis occurred only once, whereas gasserectomy is accompanied by quite a considerable percentage of these disastrous after-effects.

